# Ectopic Olfactory Receptors in Oral Health and Disease: Molecular Links Between Chemosensing, Tissue Repair, Inflammation, and Cancer

**DOI:** 10.3390/ijms27136093

**Published:** 2026-07-07

**Authors:** Jun Ohshima, Nobutake Tanaka, Masayoshi Morita, Shotaro Abe, Eriko Nakamura, Mikako Hayashi

**Affiliations:** 1 Department of Restorative Dentistry and Endodontology, Graduate School of Dentistry, The University of Osaka, Suita 565-0871, Japan; u224666j@ecs.osaka-u.ac.jp (N.T.); u368102a@alumni.osaka-u.ac.jp (M.M.); abe.shotaro.dent@osaka-u.ac.jp (S.A.); hayashi.mikako.hq@osaka-u.ac.jp (M.H.); 2 Department of Preventive Dentistry, Graduate School of Dentistry, The University of Osaka, Suita 565-0871, Japan; nakamura.eriko.dent@osaka-u.ac.jp

**Keywords:** ectopic olfactory receptor, oral mucosa, periodontitis, oral squamous cell carcinoma, microbiome–metabolite–host interaction, G protein-coupled receptor, taste dysfunction

## Abstract

Ectopic olfactory receptors (ORs) are G protein-coupled chemosensors expressed outside the olfactory epithelium, where they may couple local chemical inputs to cell-specific signaling. The oral cavity is continuously exposed to food-derived compounds, microbial metabolites, volatile organic compounds, and inflammation-associated metabolites, yet the molecular roles of oral ORs remain incompletely defined. This review critically synthesizes current evidence for OR expression and signaling in oral tissues and associated cell populations, with emphasis on ligand–receptor–signaling relationships and disease relevance. Functional OR signaling has been demonstrated in mammalian taste cells, while emerging transcriptomic studies in oral mucosa and transcriptomic/localization studies in the periodontal ligament indicate OR-related programs during tissue-specific or repair-associated states. Candidate metabolic axes, including short-chain fatty acids and lactate linked to OR51E1/OR51E2/Olfr78-related pathways in non-oral models, provide testable mechanistic hypotheses for microbiome–host communication in periodontitis and oral cancer; however, direct causal validation in oral disease models remains limited. We propose an evidence-tiered framework integrating spatial expression mapping, metabolomics-guided deorphanization, receptor perturbation, and longitudinal oral-fluid profiling. Oral ORs should currently be regarded as candidate molecular modulators and components of multimodal biomarker strategies rather than validated standalone diagnostic or therapeutic targets.

## 1. Introduction

Olfactory receptors (ORs) are G protein-coupled receptors (GPCRs) and constitute the largest gene family in the human genome, with approximately 400 functional genes in humans and over 1000 in mice [1,2,3]. In the canonical olfactory system, ORs possess seven transmembrane domains and typically activate cAMP-dependent signaling upon ligand binding [4,5]. Traditionally, ORs are recognized as receptors expressed in olfactory neurons of the nasal olfactory epithelium, responsible for odor perception through the detection of volatile chemicals [6,7,8]. However, numerous recent reports have shown that ORs are widely expressed in tissues outside the olfactory system and may also play functional roles [9,10].

OR expression has been reported in diverse organs and cells, including the skin, digestive tract, testes, heart, kidneys, blood vessels, and immune cells. These receptors are gaining attention as chemical sensors that are potentially involved in physiological functions unrelated to olfaction, such as cell migration, proliferation, apoptosis, secretion, metabolic control, and inflammatory responses [11]. System-wide expression profiling has also demonstrated that a significant number of human OR genes are transcribed in numerous tissues outside the olfactory organ [12]. Conversely, extensive transcription has been observed for some ORs, prompting discussions about leaky expression regulation and the possibility that transcripts may not always directly translate into functional protein molecules [13]. Therefore, the physiological significance of ectopic ORs cannot be generalized; it is necessary to scrutinize the expression patterns and functional roles specific to each tissue and cell type.

The oral cavity represents a chemically complex microenvironment in which food-derived compounds, volatile molecules, microbial metabolites, and host-derived inflammatory or metabolic mediators coexist. Within this environment, ectopic ORs may act as candidate GPCR chemosensors that convert local chemical cues into cell-context-dependent signaling outputs. In mature canonical mammalian olfactory sensory neurons, OR gene expression is generally organized according to a singular receptor-expression logic, often referred to as the “one neuron, one receptor” principle [14]. However, this principle should not be interpreted as an absolute rule across all chemosensory systems, species, or developmental stages, as exceptions and parallel chemosensory receptor systems have been reported, including co-expression of functional odorant receptors in insect olfactory neurons, trace amine-associated receptors (TAARs), and non-GPCR MS4A chemosensors [15,16,17,18]. In contrast, ectopic tissues may exhibit broader or more flexible receptor-expression patterns, and co-expression of multiple ORs within a single cell has been reported [19]. This co-expression raises the possibility that oral epithelial cells, fibroblasts, and immune-associated cells integrate multiple chemical inputs through receptor- and cell-state-dependent pathways, including cAMP-, Ca^2+^-, and MAPK/ERK-related signaling, thereby influencing cytokine production, proliferation, migration, barrier function, and tissue repair [10,19,20,21,22].

However, research related to ORs in the oral cavity remains in its infancy, with only fragmentary insights into tissue-specific expression patterns, cell type specificity, downstream signaling, and causal relationships with disease states. Progress has been made in oral cancer, such as the recognition of *OR7C1* as a target for oral cancer stem cells [23]. Meanwhile, research in periodontal tissues remains in its early stages, with transcriptome analysis of the periodontal ligament (PDL) regeneration process suggesting involvement of OR-related pathways [24]. More recently, transcriptomic profiling of murine buccal, gingival, and palatal mucosa has provided expression-level evidence for region-specific patterns of ectopic ORs in oral mucosal tissues [25]. However, the physiological ligands, downstream signaling outputs, and disease-related functions of these mucosal ORs remain unresolved. Notably, reviews integrating ORs across oral tissues—particularly their relationship with immune regulation and host–microbiome crosstalk, as well as their pathophysiological significance in periodontal disease and oral mucosal disorders—remain limited.

**Figure 1:**
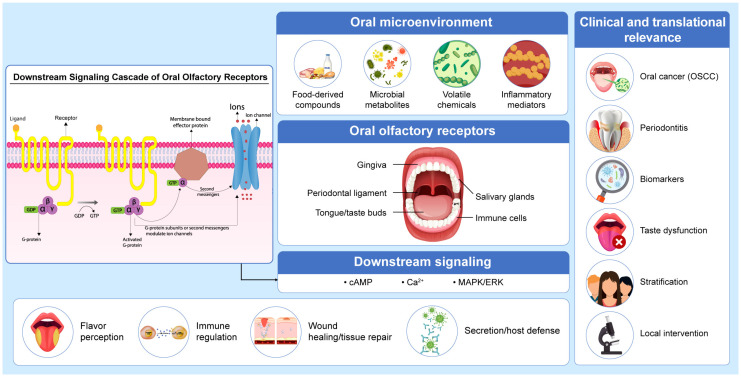
Conceptual framework of oral ORs in health and disease. This schematic illustrates a proposed framework in which ORs expressed in oral tissues and associated cell populations—including the tongue/taste buds, gingiva, periodontal ligament, salivary glands, and immune-related cells—sense local chemical cues derived from diet, microbial metabolism, volatile compounds, and inflammatory mediators. Candidate OR-associated signaling, including cAMP, Ca^2+^, and MAPK/ERK pathways, may influence oral physiology and contribute to disease-related phenotypes in oral squamous cell carcinoma, periodontal disease, and taste dysfunction. The figure also highlights potential translational implications, such as biomarker development, patient stratification, and localized intervention. This framework integrates established findings with emerging and hypothesis-generating observations.

Accordingly, this review evaluates oral ORs not merely as ectopically expressed receptor transcripts or candidate biomarkers, but as potential molecular interfaces between the local chemical microenvironment and cell-specific responses. We synthesize current evidence for OR expression and localization in oral tissues, candidate ligands derived from diet, microbial metabolism, and host inflammatory or metabolic processes, and downstream signaling outputs involving cAMP, Ca^2+^, and MAPK/ERK pathways. We then critically assess the extent to which ligand–receptor–signaling–phenotype relationships are supported in oral physiology and disease, with particular attention to periodontal inflammation and tissue remodeling, oral squamous cell carcinoma, and taste dysfunction. Finally, we identify key evidentiary gaps and propose experimental priorities for progressing from expression mapping and ligand identification to functional validation and carefully evaluated translational applications (Figure 1).

## 2. Expression of ORs in the Oral Cavity

### 2.1. Expression of ORs in the Tongue and Taste Buds

OR-related transcripts in the oral cavity were first identified through the discovery of OR-like GPCRs in tongue-associated tissues and the subsequent detection of OR-related transcripts in the tongue epithelium and taste bud-associated tissues [26,27]. In humans, olfactory-like GPCR transcripts were detected in both adult and fetal tongue tissue, suggesting OR expression in the tongue epithelium [28]. Subsequently, analysis of complementary DNA libraries derived from adult tongue surface epithelial cells has revealed multiple OR-related transcripts, including *OR7A5*/*HTPCR2*, *OR6Q1*, and *OR7C1*/*TPCR86*, further supporting the reproducibility of OR expression in the tongue surface epithelium [29].

In a follow-up study targeting mouse tongue papillae (circumvallate, foliate, and fungiform papillae), mouse orthologs of several human olfactory-like receptor (OLR) genes were detected within the papillary tissue. However, in situ analysis did not reveal clear localization to taste receptor cells within the taste buds. Based on the findings, it remained unclear whether the OR transcripts detected in the papillae were directly involved in taste reception itself [30]. Subsequently, a primary culture system of human fungiform papilla-derived cells (HBO cells) was established as an experimental platform utilizing taste bud-associated cells. Expression of taste cell-associated markers and calcium (Ca^2+^) responses to taste stimuli were demonstrated, enabling molecular expression and functional analysis of human taste bud-associated cells [31,32]. Using this platform, Malik et al. demonstrated the expression of ORs and olfactory signaling-related molecules in taste cells using OR promoter-driven green fluorescent protein transgenic mice and human HBO cells. Furthermore, they demonstrated that the Ca^2+^ response to olfactory stimuli was abolished by adenylyl cyclase inhibition or adenylyl cyclase messenger RNA (mRNA) knockdown, directly supporting functional OR signaling (at least cAMP/adenylyl cyclase-dependent) in taste bud cells [8].

These findings challenge the strict functional division model of “taste receptors in taste buds and olfactory receptors in the nasal mucosa,” suggesting that taste and smell integration may also occur at the peripheral level. As taste bud cells inherently possess GPCR-mediated chemoreceptors (e.g., for sweet, bitter, and umami) [33], OR expression within the same or adjacent cell populations may function as a localized chemosensing and response module for food-derived volatile compounds and chemical signals from the oral environment. Considering that the human tongue/oral cavity contains approximately 4600 taste buds [34], the potential physiological relevance of OR signaling in taste-associated cells warrants further investigation.

### 2.2. OR Expression in Other Oral Tissues

Compared with the tongue and taste buds, functional evidence for OR signaling in the oral epithelium and oral mucosa remains limited; however, recent transcriptomic studies have begun to provide direct expression-level evidence for ectopic ORs in oral mucosal compartments [25]. The oral mucosa is a heterogeneous tissue composed of stratified squamous epithelium, the lamina propria, glandular tissue, and immune cells; therefore, low-abundance OR transcripts may be diluted in bulk-tissue analyses. Furthermore, ORs are technically challenging to detect due to their low expression and high sequence similarity, and there are issues with antibody specificity [20,22,35]. Therefore, oral mucosal ORs should now be regarded as transcriptomically supported, at least in murine tissues, while their cellular localization, ligand responsiveness, and functional significance remain incompletely resolved.

To complement these literature-based observations, we performed a targeted survey of publicly available bulk RNA-expression resources, including the Human Protein Atlas (HPA) consensus tissue dataset and HPA-provided Genotype-Tissue Expression (GTEx) tissue and detailed tissue datasets. We focused on candidate ORs in selected oral and oral-related tissues, including the tongue, salivary gland, minor salivary gland, tonsil, and esophagus (Supplementary Table S1). These database-derived signals were generally low and variable and should be interpreted as supportive and hypothesis-generating rather than as evidence of receptor protein localization or functional OR signaling.

In salivary glands, a whole-body expression profiling study reported that mouse salivary glands are one tissue type with high OR gene expression [13], suggesting that ORs may function as chemical sensors or secretory regulators. However, experimental reports linking specific OR subtypes expressed in salivary glands with their functions remain insufficient at present.

Time-series transcriptome analysis of PDL tissue in a mouse tooth replantation model showed increased expression of multiple OR genes and enrichment of GPCR-related signaling pathways at 28 days post-replantation. In the same model, RT-qPCR and immunohistochemical analyses demonstrated increased expression and tissue localization of Olfr78 and Olfr461 in the PDL and adjacent tissues, indicating that OR-related changes are detectable at the tissue level rather than only at the pathway level. In parallel, scratch assays using human PDL fibroblasts showed time-dependent increases in the expression of several human OR genes, including *OR5B3*, *OR51E2*, *OR52N4*, and *OR10A3*, with peak expression at approximately 36 h during wound closure [24]. Together, these findings identify a candidate regeneration-associated OR transcriptional program in periodontal tissues and wound-responsive PDL fibroblasts. However, it remains unclear whether these OR changes represent markers of a repair-associated cellular state or functional regulators of periodontal repair. The endogenous ligands, receptor-specific downstream signaling pathways, and causal contributions of individual ORs to PDL regeneration or wound closure remain to be established.

Regarding PDL stem cells (PDLSCs), RNA-seq/Gene Set Enrichment Analysis of resveratrol-treated PDLSCs revealed enrichment of an OR signaling-related transcriptional program, positioning OR-related pathways within the context of crosstalk with proliferation/regeneration-related pathways, such as ERK/Wnt. Increased OR expression was also confirmed by quantitative polymerase chain reaction, indicating that OR-related transcriptional changes accompany a resveratrol-induced regenerative phenotype in PDLSCs [36]. However, whether individual ORs functionally mediate PDLSC activation or contribute directly to regenerative outcomes remains unknown. Accordingly, these findings should currently be interpreted as evidence of an OR-associated transcriptional signature within a regeneration-related cellular state, rather than as proof of OR-driven regeneration.

In the gingiva, evidence for a local chemosensory molecular environment was initially provided by spatial transcriptomic and single-cell analyses. These analyses identified the expression of chemoreception/olfaction-related molecules, such as *Omp*, *Gnal*, *Gfy*, and *Chga*, in several clusters within the gingival epithelium and submucosal layer. Cell populations, including clusters associated with Merkel cells and glandular epithelium derived from minor salivary glands, have been identified [37]. Although this study did not directly establish OR gene expression or OR-dependent signaling, it suggested the presence of spatially organized chemosensory-associated cellular niches within gingival tissue.

More recently, Pokharel et al. performed RNA sequencing of full-thickness gingival, buccal, and palatal mucosal tissues from adult mice, followed by RT-qPCR validation of selected genes. Their analysis demonstrated distinct transcriptional profiles among oral mucosal regions and showed that ectopic ORs and associated genes were nonrandomly distributed within this tissue-specific expression architecture. Several ORs exhibited tissue-biased expression patterns associated with keratinization, calcium signaling, and GPCR-related pathways [25]. These findings provide direct transcriptomic support for ectopic OR expression in murine oral mucosal compartments, including gingival tissue. Importantly, however, they do not yet establish receptor protein localization in specific cell populations, endogenous ligand–receptor relationships, OR-dependent signaling responses, or causal functions in oral mucosal homeostasis or disease.

**Figure 2:**
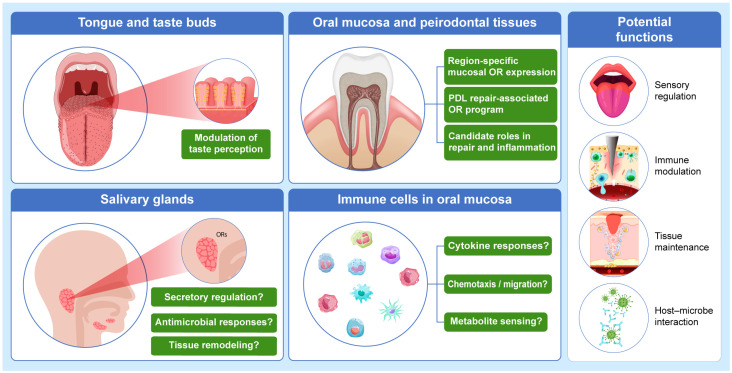
Reported expression and proposed functions of ORs across oral tissues and associated cell populations. This figure summarizes current evidence for OR expression and proposed functions across oral tissues. Functional OR signaling has been reported in taste-associated cells, supporting peripheral chemosensory responses and raising the possibility of taste or flavor modulation. In the oral mucosa, transcriptomic evidence supports region-specific ectopic OR expression in murine gingival, buccal, and palatal compartments, although functional ligand responses remain unestablished. In periodontal tissues, regeneration- and wound-associated expression of candidate ORs has been reported in the periodontal ligament and related fibroblasts, supporting a candidate repair-associated transcriptional program; however, receptor-specific functions in periodontal regeneration remain unestablished. In salivary glands, proposed functions remain largely indirect or hypothesis-generating. In immune-associated cells in the oral mucosa, putative roles include cytokine-related responses, chemotaxis, and immune–metabolic regulation, although direct evidence remains limited. Overall, the evidence suggests tissue- and context-dependent contributions of ORs to oral homeostasis and disease.

Taken together, the current evidence across oral tissues is uneven but increasingly suggests that ORs may contribute to chemosensation, immune regulation, secretion, host defense, and tissue repair in a tissue- and context-dependent manner. Functional OR signaling is best established in taste-associated cells, while transcriptomic evidence now supports region-specific OR expression in murine oral mucosa and candidate regeneration-associated OR transcriptional programs in periodontal tissues. However, receptor-specific ligand responses and causal functions in gingival and periodontal disease remain to be established (Figure 2).

## 3. Signal Transduction and Function of Oral ORs

### 3.1. OR Activation, Intracellular Signaling Mechanisms, and Ligand Identification

ORs are seven-transmembrane GPCRs. Beyond their classical role in the olfactory epithelium, they are recognized as chemical sensors regulating diverse cellular functions in non-olfactory tissues (ectopic ORs) [10,19,22]. However, OR signaling is highly dependent on ligand–receptor pairing and downstream signaling context, and the activated pathways vary depending on the cell type, receptor subtype, ligand, and co-expressed signaling molecules. To understand ORs as functional molecules in the oral cavity, it is necessary to integrate three aspects: (i) identifying the expressed ORs, (ii) identifying potential ligands that may be locally present, and (iii) associating them with intracellular signaling (e.g., cAMP/Ca^2+^/mitogen-activated protein kinase [MAPK]).

#### 3.1.1. Basic Architecture of OR Signals in the Classical Olfactory System

In classical OR signaling within olfactory neurons, ligand binding activates the OR, which then activates adenylyl cyclase (primarily AC3) via a G protein (typically Gα_olf_) [1,38,39]. This results in increased intracellular cAMP, inducing cAMP-gated (CNG) channel-mediated cation influx and membrane potential changes. Furthermore, Ca^2+^ influx contributes to signal amplification and timing (including adaptation/desensitization) via Ca^2+^-dependent processes [40]. This canonical OR–Gα_olf_–AC3–CNG pathway should be regarded as a reference model derived primarily from mature mammalian olfactory sensory neurons rather than as a universal architecture for all olfactory or ectopic chemosensory cells.

However, in tissues other than the olfactory system, this “olfactory neuron-type” system (Gα_olf_/AC3/CNG) is not necessarily fully reproduced, and in many cells, some of its components are missing. Therefore, it has been suggested that ectopic ORs may function as rewiring circuits to cell-specific G proteins and effectors, while sharing outputs, such as cAMP, Ca^2+^ elevation, and MAPK activation [10,19,22]. A relatively direct example of OR activation and intracellular signaling in the oral cavity is functional OR signaling in taste cells. Malik et al. reported that cells derived from mouse taste papillae and human fungiform papillae (HBO cells) exhibit Ca^2+^ responses to olfactory stimuli, and these responses are abolished by adenylyl cyclase inhibition and adenylyl cyclase mRNA knockdown [8]. This finding supports the notion that, at least in taste cell systems, OR activation is linked to Ca^2+^ responses via adenylyl cyclase-dependent signaling (the cAMP pathway).

#### 3.1.2. Ligand Identification (Deorphanization) Challenges and Approaches

One of the major bottlenecks in treating ORs as “functional molecules” is that many ORs remain orphan receptors [41]. However, in recent years, ligands for ectopic ORs with potential relevance to oral science have been identified. Short-chain fatty acids (SCFAs) are the best-characterized ligand class, with OR51E2 (Olfr78) activated by acetate and propionate [42]. OR51E1 (Olfr558) responds to C3–C9 carboxylic acids, particularly butyric acid, isovaleric acid, and valeric acid [43,44]. Furthermore, OR51E2 is activated by β-ionone [45,46] and lactate [47], while OR2AT4 is activated by Sandalore [40]. This demonstrates that ORs respond not only to aromatic molecules but also to endogenous metabolites.

In the search for oral ligands, prioritizing “pathological microenvironment-derived ligands” is reasonable. These consist of volatile compounds from fragrances and food, along with microbial metabolites (SCFAs, organic acids, aldehydes, volatile organic compounds [VOCs]) and host-derived metabolites (such as lactic acid and lipid peroxidation products) [10,48,49]. The oral microbiome produces a diverse array of potential ligands, including cadaverine, putrescine, volatile sulfur compounds, indole, and skatole [50,51,52]. Interactions with the oral OR repertoire remain unclear, representing a broad research area [41,53]. From this perspective, “metabolomics-based deorphanization” holds promise, identifying locally present compounds using metabolome/VOC analysis of gingival crevicular fluid (GCF), saliva, and periodontal pocket contents, and designing ligand panels based on this information.

Implementing deorphanization requires (i) a heterologous expression system that ensures cell surface expression of the receptor, (ii) designing a panel of candidate ligands, and (iii) standardizing the readout (e.g., cAMP/Ca^2+^/luciferase) [54]. Accessory factors, such as RTP1S and Ric8b, have been shown to enhance OR function [55,56], and in some cases, cell surface expression has been improved by adding an N-terminal signal peptide [57]. Resources such as Human Olfactory Data Explorer [58] and OR Database/OdorDB [59,60] are useful for designing candidate panels, but the physiological concentration range and coexisting environmental effects of these candidates in oral lesions must be separately verified.

Furthermore, cryo-electron microscopy has revealed the structure of human OR51E2 in its active state, showing that propionic acid binds within the occlusion pocket and induces structural changes in extracellular loop 3 to trigger activation [61]. This structural information, when combined with molecular dynamics simulations, has the potential to serve as a foundation for predicting novel ligands and designing specific agonists/antagonists.

**Figure 3:**
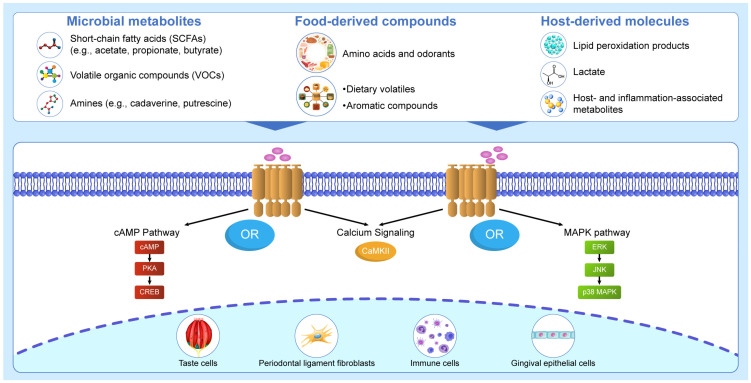
Candidate ligand–OR–signaling axes in the oral microenvironment. This figure summarizes a conceptual model of candidate ligand–OR–signaling relationships in the oral microenvironment. Potential ligands include microbial metabolites, food-derived compounds, and host-derived molecules. Representative candidate ligand–OR pairs reported in the literature include acetate/propionate–OR51E2 (Olfr78), butyrate/isovalerate–OR51E1 (Olfr558), lactate–Olfr78/OR51E2-related signaling, and Sandalore–OR2AT4. These interactions may engage cAMP, Ca^2+^, and MAPK-related signaling and influence oral cellular responses. This figure represents an evidence-informed conceptual framework.

Taken together, these findings support an evidence-informed conceptual framework in which chemically diverse ligands in the oral microenvironment may engage candidate ORs and shape downstream signaling outputs, including cAMP-, Ca^2+^-, and MAPK-related pathways, in a receptor-, cell-, and context-dependent manner (Figure 3).

### 3.2. Physiological Functions of ORs in the Oral Cavity

The oral cavity is a chemically complex environment that is in direct contact with the external environment. Its homeostasis is maintained through close coordination of the epithelium, glands, immune system, and nervous system. In such an environment, oral ORs may constitute a candidate chemosensory input layer that links local chemical cues to cell-specific responses. The next section outlines the physiological functions of oral ORs from four perspectives: (i) oral chemical sensation and taste perception, (ii) immune and metabolic responses, (iii) tissue homeostasis and regeneration, and (iv) secretion control.

#### 3.2.1. Contributions to Oral Chemical Sensation and Potential Flavor Modulation

Flavor perception arises from the integration of gustatory, olfactory, and oral somatosensory inputs [62]. Functional OR expression has been demonstrated in mammalian taste-associated cells, in which odorant-evoked Ca^2+^ responses were dependent on adenylyl cyclase signaling [8]. These findings raise the possibility that OR-dependent sensing may occur at the level of taste-associated cells and may contribute to the peripheral processing of food-derived chemical cues. However, whether oral OR signaling measurably modifies human flavor perception remains to be established. This possibility is also relevant to the study of lipid perception. CD36 and GPR120 have been implicated in oral fat sensing, while olfactory cues may contribute to the perception of fat-associated food stimuli [62]. It will therefore be important to determine whether ORs expressed in taste-associated or other oral cells respond to lipid-derived volatile compounds or metabolites and influence chemosensory responses.

In addition, periodontitis has been associated with impaired olfactory function in a clinical study [63]. However, whether this association involves altered OR expression or OR-dependent signaling within oral tissues remains unknown.

#### 3.2.2. Connections to Immune and Metabolic Responses: Inflammasomes, Cytokines, and Polarization

OR signaling functions not only in epithelial cells, but also in immune cells. Orecchioni et al. demonstrated that Olfr2 (equivalent to human OR6A2) in vascular macrophages enhances NLR family pyrin domain-containing 3 inflammasome activation and interleukin-1β production in response to endogenous ligands (e.g., octanal) [64]. In alveolar macrophages, Sandalore (OR2AT4 agonist) and citronellal (OR1A2 agonist) induce transient increases in Ca^2+^ and cAMP, reducing phagocytic activity and pro-inflammatory cytokine secretion [65]. Furthermore, Vadevoo et al. demonstrated that Olfr78, a macrophage OR, senses tumor-derived lactate and promotes M2-like macrophage polarization in coordination with Gpr132 [47]. Olfr78 immunoreactivity has also been reported in macrophages within the choroid plexus and in parenchymal microglia near the vasculature in the mouse brain, although its functional role in these central nervous system immune-cell populations remains unresolved [66].

In addition to inflammasome activation and polarization, OR signaling may also influence immune cell trafficking. In mouse CD4+ T cells, odorants cognate for T cell-expressed olfactory receptors increased intracellular cAMP and inhibited chemokine-driven chemotaxis, thereby promoting tissue retention [67], whereas in mouse pulmonary macrophages, odorant stimulation enhanced MCP-1 production and increased macrophage migration in vitro [68]. Although these findings were obtained outside the oral cavity, they raise the possibility that ectopic ORs may also modulate the chemotactic or migratory behavior of immune cells in oral inflammatory microenvironments. 

In the oral cavity (e.g., periodontal disease, oral cancer microenvironment), metabolic byproducts, such as lactic acid and aldehydes, may accumulate locally. Therefore, the possibility that ORs may modulate the inflammatory phenotype (inflammasomes, cytokines, polarization state) as upstream inputs in immune–metabolic crosstalk provides important insights into disease pathogenesis.

#### 3.2.3. OR Roles in Tissue Homeostasis and Regeneration

From the perspective of tissue homeostasis and regeneration, the PDL provides an emerging oral tissue context in which OR-related transcriptional changes are associated with repair and remodeling states [24]. In a mouse tooth replantation model, increased expression of multiple OR genes was demonstrated at 28 days post-replantation, and immunohistochemical staining confirmed Olfr78 and Olfr461 localization within PDL tissue. Furthermore, in human PDL fibroblast scratch assays, the expression of multiple human OR genes increased in a time-dependent manner as wound closure progressed. These observations support the existence of a candidate regeneration-associated OR program in the PDL, but do not establish whether OR activity is required for, or capable of promoting, periodontal wound repair.

OR2AT4 in skin keratinocytes represents a classic example of ectopic OR functional expression. OR2AT4 activation by Sandalore stimulation has been shown to promote keratinocyte proliferation and migration via Ca^2+^ signaling, thereby inducing wound healing-related responses [40]. This extraoral evidence provides a mechanistic rationale for testing whether analogous receptor-dependent repair responses occur in oral epithelial or periodontal cells. However, such an “OR-to-repair” relationship has not yet been functionally demonstrated in oral tissues. Future studies should therefore determine whether candidate ORs expressed during periodontal repair respond to locally relevant ligands and whether receptor-specific perturbation alters migration, proliferation, extracellular matrix remodeling, or periodontal regeneration outcomes.

#### 3.2.4. Secretion Control (Mediator Secretion)

In several extraoral secretory and endocrine systems, ORs can function as “secretory sensors” that convert chemical stimuli into secretory responses. Braun et al. demonstrated that olfactory stimuli induce serotonin release via ORs in the enterochromaffin cells of the human intestine [69]. Furthermore, OR activation in the enteroendocrine system has been reported to potentially involve the secretion of intestinal peptides, such as glucagon-like peptide-1, and the regulation of glucose metabolism, supporting the concept that ORs link the perception of nutrient-derived chemicals to endocrine and metabolic responses [70].

This framework may also be relevant to the oral cavity. In oral tissues, OR-dependent signaling could potentially influence the secretion of salivary components and other local mediators, including cytokines and antimicrobial molecules, through changes in intracellular Ca^2+^/cAMP signaling. However, direct evidence for such roles in the oral cavity remains limited.

### 3.3. Role of ORs in Oral Pathology

**Figure 4:**
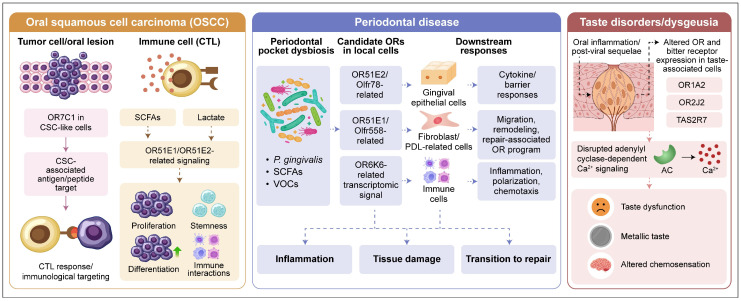
Proposed OR-related pathways in oral pathologies. This schematic summarizes proposed roles of ORs in oral squamous cell carcinoma (OSCC), periodontal disease, and taste disorders. In OSCC, OR7C1 is shown as a cancer stem cell-associated immunological target, whereas OR51E1/OR51E2-related signaling is presented as a candidate metabolite-responsive pathway. In periodontal disease, dysbiosis-associated metabolites, including *Porphyromonas gingivalis*-associated products, SCFAs, and VOCs, may provide candidate cues for OR-related pathways in gingival, immune, fibroblastic, and PDL-related cells, potentially influencing inflammation, tissue damage, and repair. In taste disorders, altered OR and bitter taste receptor expression, including OR1A2, OR2J2, and TAS2R7, may contribute to disrupted chemosensory signaling and taste dysfunction. Dashed arrows and dashed boxes indicate candidate or proposed pathways requiring further causal validation.

In oral pathologies, alterations in the microbiota, inflammatory cytokines, oxidative stress, metabolic environment, and tissue repair failure collectively cause changes in the chemical microenvironment. Therefore, it is more appropriate to understand ORs not as “causative molecules” of the pathology, but as regulatory factors that convert the pathological chemical environment into host cell responses. Changes in the ligand environment associated with pathology and the rewiring of intracellular circuits (alterations in cellular state) are particularly important because they can potentially alter OR output (e.g., cAMP/Ca^2+^/MAPK) and phenotype. The next sections focus on three representative pathologies: oral cancer, periodontal disease, and taste disorders, and the proposed OR-related pathways in these contexts are summarized schematically (Figure 4).

#### 3.3.1. ORs in Oral Squamous Cell Carcinoma (OSCC): Tumor-State Signatures, Immunological Targeting, and Candidate Metabolic Signaling

OSCC progression and therapeutic responses are increasingly understood to depend on dynamic interactions among malignant epithelial cells, stromal cells, immune cells, and the metabolic tumor microenvironment. Recent studies have highlighted immune microenvironment remodeling, cancer-associated fibroblast–macrophage crosstalk, T cell exhaustion, adipocyte-associated metabolic signaling, sphingolipid metabolism, and extracellular vesicle-mediated communication in OSCC [71,72,73]. This immune–metabolic background provides a rationale for considering ORs as candidate chemosensory or metabolite-responsive receptors that may intersect with established OSCC microenvironmental programs.

1. OR-Related Tumor-Cell-State Signatures: Evidence from Cancer Models and Relevance to OSCC

Transcriptomic studies across multiple cancer types have suggested that OR expression may reflect altered malignant-cell states rather than serving merely as incidental ectopic transcription. Using single-cell transcriptomic datasets from multiple tumor types, Kalra et al. identified combinatorial OR expression in malignant cells and showed that OR-centric signatures were associated with differentiation-related features and prognosis in breast cancer [74]. These findings provide a conceptual basis for investigating OR-related tumor-cell-state signatures in oral cancer, but do not constitute direct evidence of OSCC-specific OR dysregulation.

More directly within the oral cancer context, Yang et al. performed a bioinformatic analysis of gene-expression datasets related to oral lichen planus and OSCC and reported enrichment of the olfactory transduction pathway among disease-associated differentially expressed genes [75]. This finding suggests that olfactory-related transcriptional programs may be altered in OSCC, although it does not establish receptor-specific expression, ligand responsiveness, or causal OR function in tumor progression. Furthermore, within the oral carcinogenesis spectrum, transcriptomic profiling of proliferative verrucous leukoplakia, a high-risk oral potentially malignant disorder, identified significantly increased expression of olfactory receptor G-protein-related genes compared with conventional oral leukoplakia [76]. Together, these observations suggest that OR-related transcriptional alterations may occur in malignant or high-risk premalignant oral epithelial states; however, their receptor-specific functions and causal significance remain unresolved.

2. OR7C1 as a CSC-Associated Immunological Target

Among individual ORs investigated directly in OSCC, OR7C1 currently provides the most disease-specific evidence. Miyamoto et al. demonstrated selective expression of OR7C1 in an oral cancer stem cell (CSC)-like fraction and showed that OR7C1-derived peptides elicited cytotoxic T-lymphocyte responses, supporting its potential as an immunological target in OSCC [23]. These findings suggest that OR7C1 marks a clinically relevant tumor-cell state and may provide an antigenic target for CSC-directed immunotherapy. Consistent with the broader relevance of OR7C1 in cancer stem-like states, OR7C1 expression has also been reported as a prognostic marker in colorectal cancer, where increased expression in cancer-initiating cells was associated with enhanced tumorigenicity and poorer clinical outcomes [77]. However, this extraoral evidence does not substitute for functional validation in OSCC. Importantly, the available OSCC evidence supports OR7C1 primarily as a CSC-associated immunological target; it does not yet demonstrate that OR7C1 functions as a ligand-responsive chemosensory receptor or directly regulates CSC properties through canonical OR signaling.

3. Candidate Metabolic OR Signaling in the Tumor Microenvironment: Mechanistic Rationale from Non-Oral Models

In contrast to OR7C1, mechanistic evidence for ligand-responsive OR signaling in cancer has been derived predominantly from non-oral tumor models. The context-dependence of ORs has been well demonstrated in prostate cancer models. For example, OR51E1 has been reported to inhibit proliferation when activated by butyrate, and inhibition of proliferation has also been observed with OR51E1/OR51E2 overexpression [78]. Conversely, OR51E2 activation has been shown to promote neuroendocrine-like differentiation, potentially leading to a more malignant phenotype [79]. In addition, OR51E2/Olfr78 has been implicated in lactate sensing and macrophage polarization in a non-oral tumor microenvironment model [47]. Thus, ORs may possess both tumor-promoting and tumor-suppressing functions, making it difficult to generalize whether ORs are “good” or “bad” in the context of cancer.

These findings provide a mechanistic rationale for investigating whether metabolite-responsive OR pathways operate in the OSCC microenvironment, where tumor, stromal, and immune cell states may be influenced by local metabolic cues. However, there is currently insufficient evidence that OR51E1, OR51E2, or related metabolite-responsive ORs are expressed in functionally relevant OSCC cell populations, respond to locally available ligands, or influence OSCC progression. Accordingly, OR51E1/OR51E2 should be presented as candidate mechanistic models for future OSCC studies rather than as validated therapeutic targets in oral cancer.

4. Remaining Causal Gaps and Priorities for Validation

The available evidence, therefore, supports two distinct, non-equivalent research directions in OSCC. First, OR7C1 should be investigated as a CSC-associated antigen and potential immunological target, with particular attention to its relationship with tumor heterogeneity, treatment resistance, recurrence, and immune recognition. Second, metabolite-responsive ORs, such as OR51E1 and OR51E2, should be examined as candidate signaling receptors only after their expression, cellular localization, and ligand responsiveness are established in OSCC tissues or derived experimental models. Critical next steps include spatial and single-cell mapping of OR expression in malignant, stromal, and immune compartments; determination of receptor protein localization; measurement of candidate ligands in the OSCC microenvironment; receptor-specific perturbation combined with cAMP, Ca^2+^, and MAPK/ERK readouts; and evaluation of resulting effects on proliferation, stemness, invasion, immune interactions, and treatment response. Until such evidence is available, OR7C1 should be regarded primarily as a CSC-associated immunological candidate, whereas OR51E1/OR51E2-related pathways remain testable but unvalidated hypotheses for metabolic sensing in OSCC.

#### 3.3.2. ORs in Periodontal Disease: Translational Layers of Microbiome Metabolites and Inflammation

1. The “microbiome–metabolite–receptor” axis in the periodontal pocket

Periodontitis is now understood as a dysbiosis-driven inflammatory disease arising from reciprocal interactions between polymicrobial communities and host immune responses [80,81]. Importantly, this host–microbiome interaction is temporally dynamic rather than a fixed dysbiotic state. A recent ligature-induced mouse study showed that hepatocyte growth factor (HGF) differentially modulates the oral microbiota during early versus late experimental periodontitis, suggesting that host-derived factors can reshape microbial communities in a disease-stage-dependent manner [82]. This temporal regulation may also alter the local metabolite milieu available for OR- or GPCR-related sensing in periodontal pockets. Periodontal pathogens, such as *Porphyromonas gingivalis* (*P. gingivalis*), produce SCFAs (e.g., butyrate and propionate) within periodontal pockets [48,83]. Recent studies have demonstrated that microbial metabolites, such as SCFAs, organic acids, and VOCs, play crucial roles in regulating the pathogenesis of periodontal disease [48,84]. Dietary fibre has also been shown to modify systemic inflammatory markers, oral and faecal microbiota, and metabolomic profiles in a murine model of experimental periodontitis, although significant effects on alveolar bone loss were not observed [85].

These SCFAs are not mere metabolic byproducts; they may also function as signaling molecules for host GPCRs, particularly FFAR2/3 and specific ORs [42,43,44,86]. Incorporating ORs into this framework establishes a conceptual model whereby metabolites modulate the reactivity of epithelial, immune, and stromal cells via ORs, thereby controlling the intensity of inflammation and the timing of the transition to repair.

Specifically, Olfr78, the murine ortholog of human OR51E2, has been identified as a receptor for acetate and propionate [42], while Olfr558, related to human OR51E1, responds to C3–C9 carboxylic acids, including butyrate and isovalerate [43,44]. The detection of OR51E2/Olfr78-related and other OR-associated transcriptional changes in periodontal tissues [24] suggests the potential existence of receptor-level candidate pathways through which microbiome-derived metabolites could influence host cell function.

Indeed, metabolome analysis of GCF has shown significantly elevated SCFA concentrations at sites of periodontal disease, suggesting the potential formation of a ligand environment necessary for OR activation [87]. Several metabolites produced by oral pathogens and commensals overlap with known or candidate OR ligand classes, including acetate/propionate-responsive OR51E2/Olfr78-related pathways and carboxylic acid-responsive OR51E1/Olfr558-related pathways [42,43,44]. Periodontitis-associated anaerobes, including *P. gingivalis*, *Fusobacterium nucleatum*, and *Prevotella* species, can generate SCFAs such as acetate, propionate, butyrate, and branched-chain fatty acids [48,83,84]. In particular, *F. nucleatum* has been reported to release acetate and butyrate as major SCFA products, whereas *P. gingivalis* can produce neutrophil chemoattractants that include SCFAs [88,89]. Oral biofilms also produce lactate, VOCs, polyamines, and indole-related metabolites, some of which contribute to oral malodor and reflect anaerobic microbial metabolism [51]. Importantly, propionate and butyrate have been reported to reach low-millimolar concentrations in gingival crevicular fluid from severe periodontal sites, suggesting that local concentrations may be biologically relevant for metabolite-responsive pathways [90]. However, oral microbial metabolites or pathogen-derived metabolite mixtures have not yet been systematically tested as ligands for oral ORs and should therefore be regarded as chemically plausible candidates rather than validated endogenous OR ligands. Furthermore, transcriptome studies of periodontal lesions have revealed scattered signals from OR-related molecules. Stage-specific analysis using non-human primate models has demonstrated that OR6K6 and receptor transporter protein 3 constitute one of the signaling pathways associated with lesion progression [91]. However, as lesion tissue is multicellular, it remains unclear which cell populations express OR6K6 and contribute to its pathological function. Therefore, this evidence is currently considered hypothesis-generating.

Based on the above, it is possible that in periodontal disease, a multi-layered input involving not only “PAMP recognition” but also “metabolite sensing” determines the inflammatory output. Whether ORs are involved in this process represents an important future research question. Although this system involves bitter rather than olfactory receptors, adjacent evidence from gingival solitary chemosensory cells supports the translational plausibility of chemosensory receptor-guided intervention in periodontal tissues. In mice, topical denatonium activated gingival bitter chemosensory signaling, increased antimicrobial peptide expression, and ameliorated ligature-induced periodontitis, providing proof-of-concept that localized modulation of epithelial chemosensory pathways can reshape host–microbiome interactions in the periodontium [92].

2. OR-mediated inflammation and immunoregulation

In vitro studies have demonstrated that SCFAs have profound and complex effects on oral cells [93]. High concentrations of butyrate induce apoptosis or cytostatic responses in gingival fibroblasts [94,95], inhibit osteogenic differentiation in periodontal ligament stem cells [96], and induce ferroptosis in periodontal ligament fibroblasts [97]. Conversely, butyrate has been reported to suppress cytokine-induced intercellular adhesion molecule-1 expression in human oral squamous cell carcinoma cells, a mechanism potentially mediated by the SCFA receptor GPR43/FFAR2 rather than histone deacetylase inhibition [98].

These findings suggest that SCFAs induce opposing phenotypes—cell death, inflammation, and anti-inflammatory responses—in environments where ligand concentrations may be elevated, such as periodontal pockets. Within the gastrointestinal tract, SCFAs generally exhibit anti-inflammatory effects and promote barrier function via GPCR signaling [99]. However, high SCFA concentrations in the oral cavity have also been implicated as a potential pathogenic factor [100].

This “paradox” is easily understood when considering that multiple GPCRs, including FFAR2/3 and ORs, receive parallel inputs from metabolic products, and that downstream circuits and outputs can be reversed depending on the cell type (e.g., epithelial cells, fibroblasts, immune cells), ligand concentration range, and available G protein-coupled signaling pathways. Therefore, in the context of periodontal disease, rather than interpreting SCFAs as a “single substance,” it is necessary to verify OR-specific signaling by correlating receptors (e.g., ORs/FFARs) with cellular states.

#### 3.3.3. ORs in Taste Disorders: Connection with Oral Diseases and Inflammation

Taste disorders are multifactorial, involving zinc deficiency, medications, and neurological diseases. However, oral diseases (such as oral candidiasis, xerostomia, and inflammatory lesions) have been reported as causative or aggravating factors [101]. An epidemiological study suggested a potential association between periodontal disease and taste/smell disorders, and self-reported surveys have also documented links between periodontal disease and taste/smell dysfunction [102]. Furthermore, the potential for changes in self-reported taste and smell function before and after periodontal treatment has been evaluated [103].

Danzer et al. focused on metallic taste disorder in long-term coronavirus disease 2019 sequelae and reported reduced expression of 166 OR genes and nine bitter taste receptors in the foliate papillae of patients with metallic taste disorder. Specifically, OR1A2, OR2J2, and the bitter taste receptor TAS2R7 were identified as candidate genes associated with metallic taste perception. This suggests that disruption of receptor expression in the tongue due to viral infection may contribute to taste disorders [104].

These findings suggest that oral ORs, along with taste receptors, play a role in oral chemosensation and may modify taste experiences through changes in receptor expression and circuits associated with inflammation and infection.

## 4. Prospects for Clinical Application

Research on ORs in the oral cavity is currently in the exploratory phase. However, ORs are a receptor family with broad chemical-sensing capabilities. As GPCRs, they represent potential targets for pharmacologic intervention. They also exhibit cell state-dependent expression and functional changes, suggesting future applications in diagnostics, stratification, and therapeutic target identification. The oral cavity offers abundant samples that are easily and repeatedly collected, such as saliva, GCF, mucosal scrapings, biofilm, and biopsies. This abundance facilitates the evaluation of correlations between molecular information and local pathology, making it advantageous for clinical application [48,84,105,106].

However, the association of expression does not equate to clinical utility. Increased expression does not guarantee predictive ability for diagnosis, prognosis, or treatment response, and functional involvement does not necessarily imply safe and selective intervention [107,108]. Based on this premise, the clinical application of ORs can be summarized under the following four points.

(i) Diagnosis and biomarker evaluation: It is more realistic to evaluate multimodal indicators that integrate the expression signatures of multiple ORs, rather than a single OR, along with inflammation markers, microbiota, and metabolites (e.g., VOCs, organic acids, SCFAs) [109,110]. Currently investigated oral biomarkers include inflammatory mediators and matrix metalloproteinases, as well as microbiome-derived signatures in periodontal disease [111], metabolite and VOC profiles in oral diseases and oral cancer [112,113], and tumor-associated salivary proteins, nucleic acids, miRNAs, and extracellular vesicle-associated cargos in OSCC [114,115,116]. OR-related signatures may provide complementary information by linking local chemical cues to chemosensory or metabolite-responsive cellular states. However, their diagnostic use remains limited by low and cell type-dependent expression, incomplete ligand annotation, technical challenges in receptor-specific detection, and a lack of validated thresholds. Thus, ORs should currently be considered exploratory components of multimodal biomarker panels rather than standalone diagnostic markers. The concept that metabolic changes in cancer cells generate unique VOC profiles is supported by detection using insect olfactory systems (which read receptor response patterns, including ORs), demonstrating an indirect yet practical link between ORs and cancer diagnosis [117,118,119,120]. Within the oral cavity, integrated analysis of VOCs in the saliva, breath, and oral headspace alongside OR/chemosensory receptors is also anticipated.

(ii) Stratification and pathophenotyping: ORs may have potential clinical value, not as a binary diagnostic tool, but as an auxiliary axis for capturing the heterogeneity (e.g., immune microenvironment, metabolism, repair response) observed in OSCC, periodontal disease, and mucosal disorders [121,122,123,124,125,126]. To achieve clinical feasibility, prospective validation linking OR signatures to outcomes (e.g., recurrence, progression, treatment response, wound healing) is essential [127]. Furthermore, analytical designs that account for changes in cellular states due to disease stage and therapeutic interventions (e.g., longitudinal and repeated sampling) are desirable.

(iii) Treatment targets/interventions: Since ORs are GPCRs, therapeutic interventions, including local administration, are theoretically feasible [22]. Stimulation of OR2AT4 in the skin promotes wound healing-related processes [40], whereas studies of OR51E1 and OR51E2 in non-oral cancer models have reported context-dependent effects on proliferation and differentiation [22,45,78,79]. In OSCC, OR7C1 currently represents a candidate immunological target associated with a CSC-like state, while ligand-directed pharmacological targeting of ORs remains unvalidated. However, ORs face significant challenges, including unidentified orphan ligands, a lack of selective pharmacological tools, and dependence on cell type and pathological phase. Therefore, designing therapeutics that consider cell selectivity and localization, in addition to receptor selectivity, is essential.

(iv) Preventive, regenerative, and localized treatment design: In the oral cavity, it is highly practical to first validate “evaluable endpoints,” such as inflammation resolution, barrier repair, and wound healing promotion, as adjunctive strategies to supplement existing treatments. Specifically, we can envision a framework for optimizing intervention timing and intensity using changes in OR/metabolites related to disease activity and repair responses as indicators.

A plausible future direction is a theranostic/companion biomarker framework that uses OR (or OR signatures) to identify responsive patient cohorts and select corresponding localized interventions. Repeated collection of oral samples is possible, making it suitable for monitoring the treatment response. However, clinical implementation requires prerequisites, such as (1) assay robustness, (2) evaluation of intra-day variability, (3) reproducibility across facilities, and (4) standardization of collection and preparation. Overall, the clinical application of ORs for oral diseases remains exploratory. Nonetheless, given the accessibility of oral samples and the chemical sensing/GPCR properties of ORs, it is appropriate to position ORs not as a “single definitive marker/target,” but as a new molecular axis for stratified and supportive interventions, integrated with existing molecular, microbial, metabolic, and clinical indicators.

**Figure 5:**
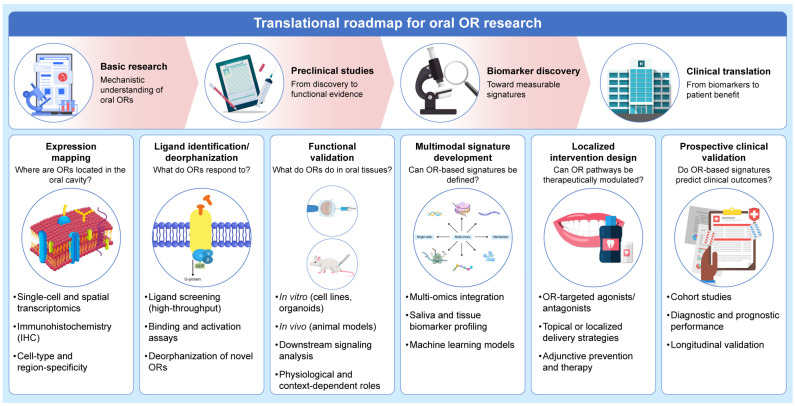
Translational pathway for oral olfactory receptor research from discovery to improved oral health outcomes. This figure summarizes a conceptual translational roadmap for oral OR research, progressing from expression mapping and ligand identification to functional validation, biomarker development, prospective clinical validation, and localized intervention design. OR-related information may be integrated with microbial, metabolic, inflammatory, imaging, and clinical data to support diagnosis, stratification, treatment monitoring, and the future development of localized therapeutic approaches in oral diseases. Because evidence remains limited and uneven across tissues and disease contexts, this figure should be interpreted as a conceptual framework.

Taken together, these lines of consideration support a conceptual translational roadmap in which oral OR research may progress from expression mapping and ligand deorphanization to functional validation, multimodal biomarker development, prospective clinical validation, and ultimately localized supportive interventions (Figure 5).

## 5. Conclusions

ORs are expressed not only in the nasal cavity but also in several oral tissues and associated cell populations, where they may constitute a candidate input layer that translates local chemical cues into cell-specific responses. Functional OR signaling has been demonstrated in the tongue and taste buds, and changes in OR expression associated with regeneration and wound response have been reported in periodontal tissues. However, the expression, localization, ligands, and downstream pathways of many ORs, including those in the oral mucosa and salivary glands, remain unknown, and verification of their causal relationships in pathological conditions remains a future challenge.

## Data Availability

No new experimental data were generated in this study. Public database-derived expression data summarized in this review are provided in Supplementary Table S1.

## Supplementary Materials

The following supporting information can be downloaded at: https://www.mdpi.com/article/10.3390/ijms27136093/s1.
